# Phosphoproteomics Meets Chemical Genetics: Approaches for Global Mapping and Deciphering the Phosphoproteome

**DOI:** 10.3390/ijms21207637

**Published:** 2020-10-15

**Authors:** Jan Jurcik, Barbara Sivakova, Ingrid Cipakova, Tomas Selicky, Erika Stupenova, Matus Jurcik, Michaela Osadska, Peter Barath, Lubos Cipak

**Affiliations:** 1 Cancer Research Institute, Biomedical Research Center, Slovak Academy of Sciences, Dubravska cesta 9, 845 05 Bratislava, Slovakia; jan.jurcik@savba.sk (J.J.); ingrid.cipakova@savba.sk (I.C.); tomas.selicky@savba.sk (T.S.); matus.jurcik@savba.sk (M.J.); michaela.osadska@savba.sk (M.O.); 2 Institute of Chemistry, Slovak Academy of Sciences, Dubravska cesta 9, 845 38 Bratislava, Slovakia; chembsiv@savba.sk (B.S.); peter.barath@savba.sk (P.B.); 3 Department of Membrane Biochemistry, Institute of Animal Biochemistry and Genetics, Centre of Biosciences, Slovak Academy of Sciences, Dubravska cesta 9, 840 05 Bratislava, Slovakia; erika.stupenova@savba.sk

**Keywords:** protein kinase, phosphorylation, chemical genetics, conditional ATP analog-sensitive mutants, mass spectrometry, phosphoproteomics

## Abstract

Protein kinases are important enzymes involved in the regulation of various cellular processes. To function properly, each protein kinase phosphorylates only a limited number of proteins among the thousands present in the cell. This provides a rapid and dynamic regulatory mechanism that controls biological functions of the proteins. Despite the importance of protein kinases, most of their substrates remain unknown. Recently, the advances in the fields of protein engineering, chemical genetics, and mass spectrometry have boosted studies on identification of bona fide substrates of protein kinases. Among the various methods in protein kinase specific substrate identification, genetically engineered protein kinases and quantitative phosphoproteomics have become promising tools. Herein, we review the current advances in the field of chemical genetics in analog-sensitive protein kinase mutants and highlight selected strategies for identifying protein kinase substrates and studying the dynamic nature of protein phosphorylation.

## 1. Introduction

Protein phosphorylation represents the most abundant type of posttranslational modification that acts as a molecular switch or rheostat for protein functions [1,2,3,4,5]. It has become widely accepted that a balance between phosphorylation and dephosphorylation, sustained by protein kinases and phosphatases, provides a dynamic regulatory mechanism to modulate protein behaviors and activities across signaling pathways [6,7]. The importance of the tight regulation of protein phosphorylation is supported by the studies showing that up to 23% of intracellular adenosine-triphosphate (ATP) may be utilized by protein kinases for phosphorylating their targets [4,8]. Interestingly, recent studies have revealed that the function of protein kinases is not limited only to phosphorylation, but that they also have roles independent of their enzymatic activities. Protein kinases might participate in the regulation of the activity of other enzymes [9,10,11,12], in the modulation of transcription processes through interactions with transcription factors [13,14,15], or function as molecular scaffolds that harmonize interactions between various components of signaling pathways [16,17,18]. Similarly, pseudokinases, which are members of the protein kinase superfamily but are their catalytically defective counterparts, function primarily through noncatalytic mechanisms. They have been shown to act as allosteric modulators of conventional protein kinases or other enzymes and proteins, thus regulating various biological processes [19,20,21]. Therefore, it is not surprising that a loss of control over protein kinases or pseudokinases has severe pathological consequences and leads to the development of various diseases, including cancers and some metabolic disorders [22,23,24,25,26,27,28,29]. Despite the central role of protein kinases in the regulation of cellular processes and their potential as therapeutic targets, only a limited fraction of protein kinases have been functionally annotated. Thus, studies on kinase–substrate relationships and the identification of protein kinase targets have become critically important for the better understanding of the regulatory functions of protein kinases.

## 2. Protein Kinases

Protein kinases belong to the great family of kinases and are responsible for the mechanism of phosphorylation. They post-translationally modify proteins by catalyzing the transfer of phosphate from ATP preferentially to the hydroxyl group of serine (Ser), threonine (Thr), or tyrosine (Tyr) residues [30]. Despite the fact that most of the effort in studying protein phosphorylation has been focused on Ser, Thr, and Tyr phosphorylation, the biological importance of other amino acids’ phosphorylation, termed as “non-canonical” phosphorylation, including histidine (His), aspartic acid (Asp), glutamic acid (Glu), arginine (Arg), cysteine (Cys), and lysine (Lys), has become evident [31,32,33]. The importance of protein phosphorylation for the regulation of biological processes is further supported by the study showing that more than two thirds of the proteome is phosphorylated. This stresses the important role of protein kinases in the regulation of cellular processes [34].

The enzymatic function of protein kinases is mediated by highly conserved protein kinase domains. In spite of the given structural conservation of these domains, protein kinases exhibit wide diversity in their ability to recognize their substrates. In general, protein kinase substrate recognition depends on the interplay between protein kinases and their regulatory proteins. Such collaboration leads to the activation or deactivation of protein kinases, to changes in their localization, or to the phosphorylation or autophosphorylation of protein kinase activation loop residues [35,36,37]. In addition, the ability of protein kinases to target their unique substrates relies on differences in their catalytic clefts that mediate the recognition of distinct phosphorylation site consensus sequences [38]. Furthermore, the non-catalytic proteins and adaptor or scaffold proteins were shown to play important roles in protein kinase substrate recognition and, in some cases, might even override the catalytic site specificity [39,40]. Recently, several excellent reviews have discussed in detail the mechanisms that cells employ to control protein kinase activities, including the substrate specificities, and recounted how protein kinases function to shape various cellular signaling networks [40,41,42]. Importantly, the phosphorylation of proteins by protein kinases also regulates protein functions by controlling their conformations. Phosphorylation can be used to balance protein–protein interactions, thus regulating protein binding and coordinating different signaling pathways. If phosphorylation occurs at or near a protein binding interface, it may directly affect the stability of the protein complex. At the same time, the phosphorylation of a site outside a protein binding interface may cause long-range conformational changes through allosteric mechanisms and alter the binding of the protein partners [43]. Similarly, the recognition of phosphorylated amino acid residues by special phospho-Ser/Thr or Tyr binding domains may release the proteins from autoinhibition and result in their activation [44].

While the function of protein kinase non-catalytic domains and the binding partners of protein kinases are well characterized, the importance of the catalytic site interactions of protein kinases still stays indistinct. This might be attributed to the unique architecture of protein kinase catalytic domains. It has been shown that protein kinase catalytic domains consist of two subdomains, mostly β-stranded N-lobe and a larger α-helical C-lobe, which are joined by a peptide strand with the cleft [45]. This peptide strand has an indispensable role for protein kinases. It has been shown that ATP binds in the cleft between the N- and C-terminal lobes of the protein kinase catalytic domain, where the adenine group of ATP is sandwiched between hydrophobic residues, and makes contact via hydrogen bonds to the hinge region [46].

While the function of the ATP-binding front pocket has been clearly defined in both catalysis and ATP binding [47,48], the role of hydrophobic back pockets remained a mystery until finding that it might directly participate in the regulation of the protein kinase catalytic function through controlling the access of ATP. Furthermore, it has been shown that the access of ATP to the protein kinase catalytic site is tightly controlled by two specific residues: a conserved lysine residue and a specific amino acid residue, named a “gatekeeper” residue. The “gatekeeper” residue has been shown to contribute to the hydrophobic spine of the kinase domain and to play an important role in promoting the protein kinase enzymatic activity [49,50,51].

Following studies utilizing the conserved lysine residue of protein kinases to modify the catalytic activity of protein kinases revealed that the replacement of the conserved lysine residue by other amino acids leads to catalytically inactive protein kinase-dead mutants [20,52,53,54,55]. This approach has been successfully employed to generate several non-essential protein kinase mutants. These mutants together with their deletion mutants helped to reveal the biological functions of some non-essential protein kinases [56,57]. The protein kinases whose activities are indispensable for cell division, and as such are considered as essential, resisted such substitutions. Therefore, analysis of biological functions of essential protein kinases stayed fully dependent on synthesis of specific protein kinase inhibitors or on isolation of their viable conditional mutants. Regardless of the power of chemical synthesis, most of the synthesized protein kinase inhibitors lack sufficient specificities. Additionally, conditions which are used to inhibit the activity of isolated protein kinase mutants (e.g., higher temperature for the inactivation of temperature-sensitive protein kinase mutants), usually lead to deviated changes in the phosphoproteome. This masks the exact functions of the analyzed protein kinases. Furthermore, continuous perturbation in protein kinase activity makes bona fide substrate identification highly unreliable. Unarguably, the permanent loss of particular protein kinase activity forces cells to substitute the limited phosphorylation of affected proteins by other protein kinases, thus hampering the identification of protein kinase substrates.

## 3. Analog-Sensitive Kinase Technology

To overcome the above-mentioned limitations, Shokat and colleagues have developed a sophisticated chemical genetics approach known as a “bump-and-hole” or “analog-sensitive” strategy that allows for the rapid, reversible, and highly specific inhibition of catalytic activity of the engineered protein kinases. Generally, in this bump-and-hole approach, a protein–protein or protein–ligand interface is altered in a way such that the engineered interface remains biochemically competent but orthogonal to the wild type. Altering either a steric or electrostatic component of non-covalent interactions at the protein–ligand interface allows the designed cofactor or inhibitor to bind only with the engineered protein through paired interactions but not with the native counterpart. Achieving orthogonality through steric or shape complementarity makes the bump-and-hole strategy one of the most powerful allele-specific chemical genetics strategies [58,59].

**Figure 1:**
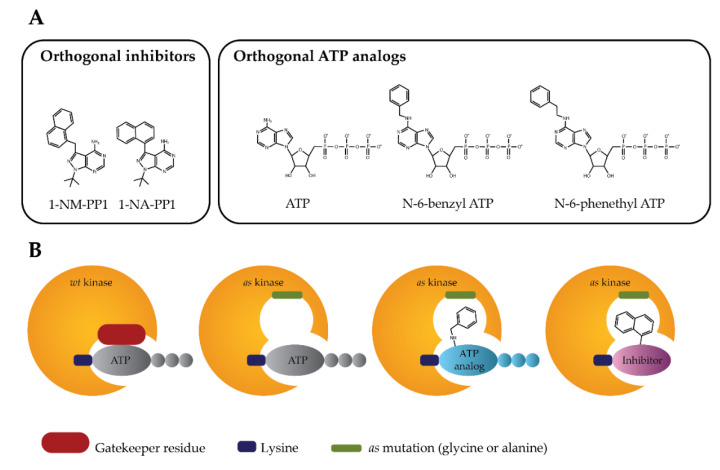
Analog-sensitive kinase technology. (**A**) Structures of orthogonal inhibitors or orthogonal ATP analogs designed to inhibit analog-sensitive (*as*) protein kinase mutants. (**B**) A schematic diagram of the analog-sensitive protein kinase technology illustrates the specific binding of bulky orthogonal ATP analog or orthogonal inhibitor to mutant *as* protein kinases. 1-NM-PP1: 4-Amino-1-tert-butyl-3-(1′-naphthylmethyl)pyrazolo[3,4-d]pyrimidine; 1-NA-PP1: 4-Amino-1-tert-butyl-3-(1′-naphthyl)pyrazolo[3,4-d]pyrimidine.

The analog-sensitive kinase strategy (Figure 1) is based on the finding that protein kinases with small “gatekeeper” residues, such as Src-family kinases, allow the binding of molecules with large bulky groups, such as the C3-tolyl ring of PP1 (1-(Tert-Butyl)-3-(p-tolyl)-1H-pyrazolo[3,4-d]pyrimidin-4-amine). This means that any protein kinase might become sensitive to this type of inhibitor, if the native large “gatekeeper” residue is substituted with smaller amino acids [49,60,61,62,63,64]. Combining the specific space-creating mutations in the ATP-binding pocket (hole) designed by amino acid substitutions of the conserved large hydrophobic “gatekeeper” residues of protein kinase (e.g., methionine (Met), leucine (Leu), phenylalanine (Phe), or Thr) with amino acid residues bearing smaller side chains (e.g., glycine (Gly) or alanine (Ala)) and the specificity of the bulky orthogonal ATP-competitive inhibitors to complement the shape of the designed mutant ATP pockets (bearing complementary bump), they established the approach referred to as analog-sensitive kinase technology or a strategy for the generation of the analog-sensitive (*as*) protein kinase mutants [65].

Contrary to the conserved Lys residue (Lys72 equivalent), which is involved in the binding of ATP through its α- and β-phosphates, whose substitution results in catalytically inactive protein kinase-dead mutants [1,52,66], the “gatekeeper” residues have been shown to be easily interchangeable. It has been demonstrated that potent and specific protein kinase inhibitors can be readily identified by testing various *as* protein kinase mutants for a panel of PP1 derivatives with enlarged C3 substituents [67,68]. Importantly, the mutagenesis of “gatekeeper” residues only slightly modifies the ATP binding, with indiscernible effects on protein kinase activities. Thus, analog-sensitive kinase technology might be applicable for nearly all protein kinases for which “gatekeeper” residue is defined [69]. The biological significance of “gatekeeper” residues for protein kinase activities is also supported by studies showing that these residues are often mutated in protein kinases resistant to clinically relevant protein kinase inhibitors [46,70,71,72,73]. In such protein kinases, the point mutations of the “gatekeeper” residues lead to perturbations in the hydrophobic pocket located at the back of the ATP binding site. As an example, the loss of affinity of BCR-ABL kinase to the inhibitor is a result of a steric clash between the inhibitor and the mutated “gatekeeper” residue [74], while in the case of the EGFR the “gatekeeper” mutation elicits a significant increase in the affinity of mutant protein kinases for ATP, thus reducing the affinity for the kinase inhibitor [75]. The importance of “gatekeeper” residues is further supported by studies showing that the identification of functional “gatekeeper” residues and the creation of conditional *as* protein kinase mutants represent a powerful tool for unravelling the complexity of protein kinase functions [49,76,77,78,79,80,81].

### Constructing Conditional Analog-Sensitive Protein Kinase Mutants

In general, the substitution of a gatekeeper residue in close proximity to the N-6-position of ATP, either by Gly (further referred as *as1* mutation) or Ala (further referred as *as2* mutation), allows the orthogonal ATP analogs or the orthogonal inhibitors with bulky substituents (Figure 1a) to be accommodated in an enlarged space of the active site of engineered *as* protein kinases (Figure 1b) [49,69,82].

The *as1* mutation provides the largest expansion of the ATP-binding pocket, thus maximizing the difference in sensitivity between the *as1* protein kinase mutant and wild-type protein kinases. However, in cases when the *as1* protein kinase mutant suffers compromised activity, the *as2* mutation can be employed to increase the activity of mutant protein kinase while maintaining its sensitivity to the orthogonal ATP analogs or the orthogonal inhibitors. In most cases, the space-creating mutation of the “gatekeeper” residue is functionally silent and does not interfere with the function of protein kinase [68]. Sometimes the substitution of the “gatekeeper” residue might lead to a loss of protein kinase activity, or the protein kinase mutant is not suited for sensing to the orthogonal ATP analogs or the orthogonal inhibitors. Then, compensatory mutations which rescue the activity of hypomorphic *as1* or *as2* protein kinases and further adapt the ATP binding pocket, making it more accessible to orthogonal ATP analogs or orthogonal inhibitors, are necessary (Table 1) [82,83,84,85,86,87,88,89,90,91,92,93,94].

Generally, the restoration of the disordered catalytic activity of *as* protein kinase mutants is realized by mutating the specific amino acid residue immediately adjacent to the catalytic Lys (K *+1*) in subdomain II of protein kinase to the branched aliphatic amino acid, such as valine (Val) or isoleucine (Ile). This approach was successfully applied to create the GRK2-*as5* protein kinase mutant with a full functionality and analog sensitivity, as compared with wild-type GRK2 protein kinase or catalytically inactive GRK2-*as1* protein kinase mutant [83]. A similar approach was applied to restore the protein kinase activity of both Cdc5-*as* and MEKK1-*as* protein kinase mutants to near wild-type level [84]. Moreover, the mutation of amino acid residue at *−1* of the DFG motif in subdomain VII of protein kinase to Ala can further enhance the sensitivity conferred by the canonical *as1* and *as2* mutations generating *as3* and *as4* protein kinase mutants. If, in the sequence of *−1* of the DFG motif, the amino acid is other than Gly or Ala, the introduction of Ala at this position may greatly enhance the sensitivity of protein kinase mutant to the inhibitor. In relation to this, it was shown that amino acid at *−1* position of the DFG motif plays an important role in the PP1 binding to the Src family tyrosine kinase Hck [85]. Similarly, the substitution of Thr710 in the budding yeast PAK Cla4p-*as2* protein kinase mutant to Ala or the substitution of Cys175 in the budding yeast Prk1-*as1* protein kinase mutant to Ala greatly enhances the sensitivity of the Cla4-*as4* or Prk1-*as3* protein kinase mutants, respectively [86,87]. Similar substitutions were also used to create *as* mutants of Cdc7 and JNK protein kinases. While Cdc7-*as* protein kinase mutant resembled Cdc7-*ts* protein kinase mutant at the restrictive temperature, the JNK-*as* protein kinase mutant was able to accommodate [γ-^32^P]-N-6-phenethyl ATP without affecting the protein kinase substrate recognition [88,89]. Additionally, the substitution of amino acid residue at *−1* position of the DFG motif (Thr244) in the *as1* background to Gly increases the sensitivity of Ipl1-*as6* protein kinase mutant to 1-NA-PP1 [84,90,91]. The analog-sensitizing amino acid residues are also positioned in protein kinase subdomains IV and VIb. Recently, it was found that the substitution of Leu to Val in the protein kinase subdomain IV of Aurora A, Aurora B, and Alk7 leads to the creation of their *as7* protein kinase mutants, which are sensitive to both orthogonal ATP analogues as well as to orthogonal inhibitors [89,90,92]. Subsequently, it was shown that additional mutations at the bottom of the ATP-binding pocket, represented by the substitution of Met to Phe residue at position *+2* relative to the HRDLKxxN motif in the subdomain VIb of protein kinase, further sensitize the *plo1-as8*, *orb5-as8*, *orb6-as9*, and *wee1-as8* protein kinase mutants to orthogonal inhibitors [93,94]. Importantly, protein kinases which are intolerant to analog-sensitive kinase technology might be subjected to a chemical-genetic approach based on a Cys-gatekeeper mutation and non-covalent, type II mode of kinase inhibition that targets the inactive “DFG-out” kinase conformation [95]. Recently, several elegant studies employed analog-sensitive mutants of CDKL5 and some other CDKs to better characterize the function of these protein kinases. The studies suggested that the identified substrates of CDKL5 kinase might be important biomarkers in the diagnosis and treatment of CDKL5 disorders. Additionally, the CDK12 and CDK13 have been identified as fundamental regulators of the global PolII processivity and transcription elongation [96,97,98,99,100].

In the case that suggested amino acids substitutions do not lead to the restoration of the disordered catalytic activity of mutant *as* protein kinases and difficulties persist, the functionality of the *as* protein kinase mutants might be improved by performing error-prone PCR, which induces additional mutations to the protein kinase subdomains, potentially suppressing problematic hypomorphic phenotypes. This strategy was successfully used to eliminate the undesirable hypomorphic phenotype of the *cdc2-as1* protein kinase mutant. The suppressor Lys79Glu mutation in *cdc2-asM17* rescued all the phenotypic defects of *cdc2-as1*, but the in vitro kinase assay revealed that Cdc2-*asM17* is in fact less active than Cdc2-*wt* in the absence of an inhibitor. Although it had a lower activity, the *cdc2-asM17* mutant helped to reveal an important role of Cdc2/Cdk1 in SAC maintenance, and suggested the dispensability of Cdc2/Cdk1 for the localization of shugoshin in meiosis [79].

Despite the fact that the chemical genetic strategy has become a powerful tool to probe protein kinase targets and that more than 150 various conditional *as* protein kinase mutants have been created so far, there are also some limitations of this approach. The most critical limitation is the genetic manipulation of ATP binding pocket, which might lead to slightly attenuated catalytic activities of the mutant protein kinases and potentially lower the affinity for ATP of the created *as* protein kinase mutants. Additionally, some difficulties are also associated with the intracellular delivery of bulky ATP analogues and the higher catalytic efficiency of *as* protein kinase mutants when the bulky ATP analogue is used [101]. However, the possibility of the rapid and reversible inactivation of *as* protein kinase mutants in a dose-dependent manner clearly surpasses these limits. The conserved nature of the above-mentioned “critical” residues in the particular protein kinase subdomains renders the chemical genetic strategy the most powerful tool in designing the protein kinase mutants. Most importantly, conditional *as* protein kinase mutants might allow us to reveal mostly unexplored and hidden functions of thus-far uncharacterized protein kinases.

## 4. Strategies for the Identification of the Protein Kinase Targets and Studying the Dynamics of Protein Phosphorylation

In spite of the significance of protein kinases for the regulation of protein functions, still about 80% of protein kinases do not have annotated substrates. Evenly, more than 95% of identified phosphorylation sites have no known protein kinase or biological function [102]. Therefore, it is plausible that the mass spectrometry-based identification of bona fide protein kinase substrates will bring light to the mechanisms involved in the dynamic regulation of cellular processes.

**Figure 2:**
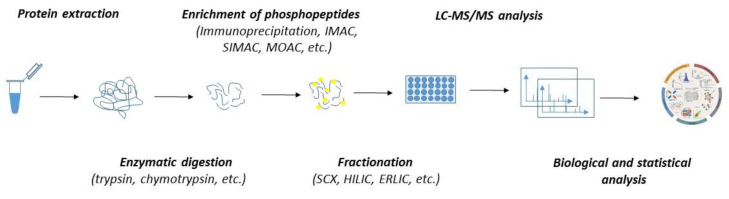
The workflow of MS-based quantitative phosphoproteomics. Both label-free and labelled samples might be subjected to protein extraction and enzymatic digestion by multiple proteases. The peptides can either be subjected to fractionation prior to the enrichment of phosphopeptides or directly subjected to phosphopeptide enrichment and fractionation applied after the phosphopeptide enrichment. Phosphopeptides are analyzed by LC-MS/MS. Mass spectrometry datasets are processed with software to generate the identification list and assign and quantitate the phosphorylation sites.

In the past few decades, the advancement in mass spectrometry techniques has enabled us to extend global proteomic studies into the identification and characterization of the biological role of various posttranslational modifications, including protein phosphorylation [103,104,105,106]. Currently, several thousand phosphosites might be routinely identified within a single sample. Despite the great robustness, phosphoproteomics is significantly limited by the variability and substoichiometric abundance of phosphoproteins. As such, the detection of phosphorylation by mass spectrometry must include—except for the common steps of regular proteomics (such as protein extraction and enzymatic digestion)—additional steps stabilizing the phosphosites and ensuring the enrichment of the sample with phosphoproteins or phosphopeptides (Figure 2).

To stabilize and preserve the native level of phosphosites, strong denaturants, such as urea and protease and phosphatase inhibitors, are commonly applied during protein extraction. Since the abundance of phosphorylated proteins is estimated to be one to two orders of magnitude lower than that of non-phosphorylated proteins, the enrichment step must be included. Currently, there are various approaches for phosphopeptide enrichment, which include strategies such as immunoprecipitation with highly selective antibodies; immobilized metal affinity chromatography (IMAC); metal oxide affinity chromatography (MOAC) with enhancers—e.g., titanium oxide (TiO_2_) or zirconium oxide (ZrO_2_); and more [107]. Despite the fact that these techniques are being extensively applied for phosphopeptide enrichments, they have some caveats which must be considered before sample processing. For example, IMAC, which favors multiply phosphorylated peptides, has a low tolerance towards buffers or salts in biological samples, and its unspecific binding towards acidic peptides might compromise its specificity. Similarly, the limits of MOAC, which, on the other hand, favors mono-phosphorylated peptides, are also related to its lower specificity due to its potential binding to acidic peptides. To overcome the limits, these approaches were integrated into a single workflow known as sequential elution from IMAC (SIMAC). Recently, polymer-based metal-ion affinity capture (PolyMAC) has emerged as a novel strategy for capturing phosphorylated peptides. It is based on water-soluble, globular dendrimers multifunctionalized with metal ions (such as Ti^4+^ and Fe^3+^). PolyMAC displayed incredible reproducibility and selectivity and high recovery rates of phosphopeptides even from complex protein mixtures [108].

Given the high amount of nonphosphorylated peptides and the large dynamic range of the phosphoproteome, separation procedures either prior to or after phosphopeptide enrichment must be included to enhance the identification of phosphopeptides. These strategies are represented by strong cation exchange (SCX) chromatography, hydrophilic interaction liquid chromatography (HILIC), and electrostatic repulsion hydrophilic interaction chromatography (ERLIC) [33,104,109,110]. As massive amounts of data are produced during phosphoproteomics analysis, various computational tools ranging from comparative statistics through to more sophisticated bioinformatics assessment have been developed collaterally to identify and quantitate phosphopeptides [111,112,113]. The choice of computational tools depends on whether the analyzed samples are labelled or label-free. Labelled quantitative phosphoproteomics, which enables the multiplexing of labelled samples, requires more experimental effort, but the data analysis is quite simple. On the other hand, label-free phosphoproteomics, which allows an unlimited number of analyzed samples to be compared, suffers from increased data variability and the overall instrument and computational time.

The pivotal study that deciphered the dynamic nature of the phosphoproteome coupled a labelling technique known as stable isotope labelling by amino acids in cell culture (SILAC) with global mass-spectrometry analysis (MS). This study showed that the phosphorylation status of the proteins varies over time [30]. Following works in various biological contexts further expanded the view of the dynamic nature of protein phosphorylation and disclosed that protein kinases actually form highly interconnected networks [114,115,116]. This established the phosphoproteome as an intricately entangled network with phenomenal complexity. The concept of the phosphoproteome being tightly controlled by protein kinases and phosphatases has become fundamental for understanding the complexity of protein phosphorylation [106,117].

In general, the approaches to identify protein kinase targets are divided into two categories: the direct identification of protein kinase substrates and the indirect phosphoproteomics-based strategy. Both approaches have their advantages and limitations. While the direct techniques for identifying the protein kinase targets search for their interacting partners by employing in vitro peptide or protein arrays on either cell lysates or permeabilized cells, the indirect techniques employ live cells.

### 4.1. Direct Strategies for Identification of the Protein Kinase Targets

The basic approach to screen protein kinase substrates is to directly characterize the protein kinase interactomes. The strategy is based on the prediction that protein kinase–substrate pairs can be co-purified when interacting. However, the interactions of protein kinases with their substrates are transient and once the proteins are phosphorylated, the interactions are lost. Additionally, protein kinase interactomes are distorted by high false positive identifications. Compared to real protein kinase substrates, there are numerous proteins interacting with protein kinases physiologically, including the components of protein complexes or the adaptor proteins. Thus, the identification of protein kinase substrates through the isolation of protein kinase interactomes has its limits [118,119]. Despite this, several studies demonstrated that bona fide protein kinase substrates might be successfully identified through the affinity isolation of protein kinase interactomes [120,121,122].

Another direct method to determine protein kinase substrates is the in vitro kinase assay in which the purified protein kinase is incubated with its predicted substrates in the presence of ATP [123]. The approach is functional with almost all protein kinases regardless of the substrates, as long as the protein kinases are in their active forms. However, the in vitro kinase assay has several limitations. First is that phosphorylation in vitro may differ from what takes place in vivo, and there is necessity to use higher concentration of purified protein kinase to overcome its usually decreased activity compared to in vivo. Second, the use of protein kinases outside the cells often leads to a loss of specificity. Third, the purification of an active protein kinase is sometimes challenging, as many protein kinases require adaptors or scaffold proteins to regulate their activity and substrate specificity. Finally, the in vitro kinase assay has a low throughput and is quite laborious, since each protein kinase is assayed with one potential substrate at a time. Importantly, similar as for the above discussed direct protein kinase-substrate identification, the identified phosphorylations found in vitro must be validated by in vivo studies [124]. Thus, the identification of protein kinase substrates and characterization of protein kinase functions require more robust strategies. Recently, several advanced high-throughput strategies for the direct identification of protein kinase substrates have been developed. These approaches, represented by protein microarrays, kinase-interacting substrate screening (KISS), heavy ATP kinase assay combined with quantitative mass spectrometry (HAKA-MS), or the analogue-sensitive kinase approach (ASKA), allowed us, despite many false positive identifications, to screen and track down some novel biologically relevant substrates of protein kinases [8,65,101,125,126,127,128].

### 4.2. Indirect Strategies for Identification of the Protein Kinase Targets

Contrary to the approaches for the direct identification of protein kinase substrates, indirect phosphoproteomics-based techniques allow us to study protein kinases and their substrates in their native in vivo environment. It should be noted that each of these methods identifies both direct and indirect protein kinase targets. Most importantly, the indirect phosphoproteomics-based approaches allow to explore the dynamic nature of protein phosphorylations (Table 2).

The foundational indirect in vivo phosphoproteomics-based approach, known as phosphate inhibitor and kinase inhibitor substrate screening (PIKISS), has been originally developed to validate the candidate substrate groups identified by direct KISS approach [126]. The PIKISS technique employs the treatment of cells with phosphatase inhibitors combined with kinase-specific inhibitors [129]. While the phosphatase inhibitor treatment of cells enhances the phosphorylation levels of proteins, including those phosphorylated under specific conditions, such as requisite priming phosphorylation, the treatment of cells with specific protein kinase inhibitors allows the detection of phosphorylation by inhibited protein kinase. Additionally, the sensitivity of PIKISS might be further enhanced by the phosphopeptide enrichment step using proteins or domains with an affinity to physiologically regulated phosphoproteins. For example, 14-3-3 proteins are well-known for their ability to interact with a variety of cellular proteins containing phosphorylated Ser/Thr residues [130,131]. Alternatively, more traditional biochemical methods involving immunoaffinity purification are also in use for the enrichment of phosphoproteins or phosphopeptides. Additionally, the antibody-based capture methods using highly selective antibodies might be adopted to investigate diverse components of signaling cascades [132,133]. Proteins and domains with an affinity for phosphoproteins might be also used to enrich the samples for components of signal transduction cascades [131,134,135]. Recently improved PIKISS, renamed kinase-oriented substrate screening (KIOSS), utilized phosphoprotein-binding modules, such as 14-3-3 proteins, the pin1-WW domain or the Chk2-FHA domain as biological filters to identify substrate candidates for PKA, PKC, MAPK, and Rho-kinase. The KIOSS technique was successfully implemented to analyze the phosphorylation downstream of D1R, NMDAR, adenosine A2a receptor, PKA, PKC, MAPK, and Rho-kinase [136]. As such, the principles of the KIOSS strategy might become applicable to any conditional *as* protein kinase mutant for which specific protein kinase inhibitors are available.

Other tools to identify protein kinase substrates are based on the comparative quantitative phosphoproteomics of intact cells treated with a specific protein kinase inhibitor. The principle of these approaches resides in the accurate quantification of the identified phosphopeptides after the treatment of cells with a specific protein kinase inhibitor. This can be done by the calculation of the ratios of peptides and phosphopeptides between conditions, or by the absolute calculation of phosphopeptides within conditions [137]. To get the most precise quantification of changes in phosphorylation, the cells are allowed to metabolically incorporate isotope-labelled amino acids using stable isotope labelling with amino acids in cell culture (SILAC). A mixture of “heavy” (heavy isotope amino acids labelled cells) and “light” (regularly growing cells) cells, concomitantly treated with either vehicle or a protein kinase inhibitor, might be directly lysed, trypsinized, enriched for phosphopeptides, and analyzed. Mixing the SILAC samples diminishes the false positives arising due to experimental errors of individually processed samples. As a result, in the MS spectra each peptide appears as a doublet with distinct mass differences. The differential abundances between the analyzed samples are calculated directly by comparing the intensity of differences for the pairs of isotope labelling peaks in MS. SILAC-combined mass spectrometry analysis (SILAC-MS) has been successfully used to screen various drug targets [138], analyze differences in post-translational modifications [139,140], follow dynamic changes in meiotic proteomes [141,142], and search for key factors of the signal pathways [143], and has been also applied to reveal the involvement of protein kinases in cell cycle regulation [81,144]. Despite the fact that classical SILAC-MS is a highly reliable method and provides a very high accuracy, its main shortage is a limited number of variations for analyzed cells, which makes this technique applicable mainly for protein quantification. To enlarge the practicability of SILAC, several variations of SILAC were developed—e.g., spike-in SILAC-MS, super-SILAC-MS, or triple-SILAC-MS [145,146,147]. For example, the spike-in SILAC-MS was developed to label samples separately from the biological experiments. The unlabeled samples are then combined with the SILAC standard, and each of the combined samples is analyzed separately. The variability between the experimental samples is calculated as the “ratio of ratios”, where the ratio of one sample relative to the SILAC standard is divided by the ratio of the other samples relative to the SILAC standard. A similar strategy is used also in triple-SILAC or super-SILAC, which are broadened to three or five SILAC-labelled samples, thus allowing an even more precise quantification of the protein phosphorylation. Except for SILAC labelling, which incorporates isotopically labelled amino acids in vivo, the samples might be subjected to chemical labelling by employing labelling techniques, such as isobaric tag for relative and absolute quantitation (iTRAQ) [148], isotope coded affinity tag (ICAT) [149], phosphoprotein isotope-coded tags (PhIAT, PhIST) [150,151], or isobaric tandem mass tags (TMT) labelling [152,153]. The known mass difference between labelled and unlabeled phosphopeptides allows us to distinguish between various biological conditions. However, labelling approaches mostly suffer from the finite number of labels/tags that can be used simultaneously. This limits total sum of conditions and samples that can be successfully compared.

Regardless of the fact that labelling approaches represent powerful strategies for the profiling of protein kinase targets and the unbiased analysis of the dynamics of protein phosphorylation, label-free quantitative (LFQ) phosphoproteomics is currently becoming the golden platform. LFQ avoids the limits of metabolic and chemical labelling and allows a theoretically unlimited number of samples to be compared. The power of the LFQ approach was clearly demonstrated by the description of the ultradeep human phosphoproteome, where more than 50,000 phosphopeptides and more than 38,000 phosphosites were identified [34]. At the same time, this work demonstrated one of the drawbacks of LFQ, as the overall instrument time of the analysis reached about 40 days.

One of the main objections to LFQ is that each sample has to be processed and measured independently. This decreases its reproducibility. The variability of LFQ might be also worsened, as various processing steps are required for sample preparation prior to MS analysis. As such, appropriate caution must be taken during sample preparation to decrease the variability in the detected phosphopeptides. Importantly, as the relative amounts of phosphopeptides may not be representative of their in vivo level, it is also necessary to check if the signals of the detected phosphopeptides are proportional to their concentration in the original sample. As a rule of thumb, label-free quantification requires more measurements or repeats to achieve acceptable levels of statistical significance. The workflow of LFQ phosphoproteomics consists of three distinct steps: sample preparation, MS analysis, and computational data analysis. In general, the enzymatically digested samples are individually desalted using reversed-phase solid phase extraction and enriched for phosphopeptides using an appropriate phosphopeptide enrichment method. The enriched fractions are then analyzed one by one using nLC-MS/MS, following reconstitution in buffer containing an array of internal standard peptides. The obtained raw MS data are processed to generate peak lists further analyzed by a search engine (e.g., Mascot™) to obtain the phosphopeptide identification data. Following this, the acquired data are used to create a database of all the phosphopeptide identifications from individually analyzed samples. From the retention time (*t*_R_), charge (*z*), and mass-to-charge ratio (*m*/*z*) data for each phosphopeptide, and by predicting the *t*_R_ values of each peptide in the particular sample, the individual chromatograms are effectively aligned using the *t*_R_ of spiked peptide standards or common ions. This allows each phosphopeptide to be quantified from its MS1 precursor ion signal. It should be noted that, although not every phosphopeptide may have been subjected to MS2 fragmentation in each sample, it still remains quantifiable [152,154,155,156].

Combining identification and quantitation gives the LFQ approach a great advantage over labelling methodologies. The data obtained in label-free proteomic experiments that were designed solely for protein and/or phosphopeptide identification can be further processed in the LFQ pipeline, providing additional quantitative data. However, great precaution needs to be taken to ensure the reproducibility of the sample preparation and data acquisition of the individual datasets. Label-free proteomic strategies will therefore remain one of the main approaches to address quantitation challenges in proteomics and phosphoproteomics.

## 5. Perspectives

Protein phosphorylation is a major and essential post-translational modification that plays an important regulatory role in various cellular processes. Although several protein kinases have been well characterized and demonstrated to be physiologically important for the regulation of various protein functions, the vast majority of protein kinases remain uncharacterized. The recent advancement in the chemical genetics strategy of conditional *as* protein kinase mutants and the rapid improvements in mass-spectrometry-based phosphoproteomics offer a great opportunity to finally uncover the regulatory functions of thus-far uncharacterized protein kinases and identify their hidden substrates. As the chemical genetic strategy can be applied to almost any protein kinase, the phosphoproteomic analysis of currently available or newly created conditional *as* protein kinase mutants would definitely unveil biologically relevant protein kinase–substrate relationships. Additionally, the phosphoproteomic analysis of *as* protein kinase mutants could reveal the fundamental principles implicated in the regulation of phosphoproteome dynamics and might help us to understand the spatiotemporal regulation of cellular processes.

## Figures and Tables

**Table 1:** Summary of each type of analog-sensitizing substitution identifiable in particular protein kinase subdomains that can be employed to create analog-sensitive (*as*) protein kinase mutants. The canonical sensitizing mutations convert a conserved bulky “gatekeeper” residue into either Gly (*as1* protein kinase mutant) or Ala (*as2* protein kinase mutant).

“Gatekeeper” Mutation	Conserved Amino Acid/Motif Suited for Analog-Sensitizing Substitution	Analog-Sensitizing Substitution	Protein Kinase Mutant
Gly	-	-	*as1*
Ala			*as2*
Gly	*−1* of DFG (subdomain VII)	Ala	*as3*
Ala		Ala	*as4*
Gly		Gly	*as6*
Gly	Lys *+1* (subdomain II)	Val/Ile	*as5*
Gly	Leu (subdomain IV)	Val	*as7*
Gly	HRDLKxxN *+2* (subdomain VIb)	Phe	*as8*
Ala		Phe	*as9*

**Table 2:** Summary of the basic characteristics of selected indirect phosphoproteomics-based techniques developed to identify protein kinase targets and characterize the dynamics of protein phosphorylation.

Technique	Labelling	Advantages	Limitations
Phosphatase inhibitor and kinase inhibitor substrate screening (PIKISS)	n.a. in vitro	Specific enrichment of relevant phosphorylated proteins.	Inhibitor might elicit off-target effects. Up to 4 samples can be compared.
Kinase-oriented substrate screening (KIOSS)			
Stable isotope labelling with amino acids in cell culture followed by quantitative phosphoproteomics (SILAC-MS)	in vivo ex vivo	High accuracy. Allows us to study the dynamics of phosphorylation. Global-scale identification of protein kinase targets.	Inhibitor might induce off-target effects. Up to 5 samples can be compared.
Label-free quantitative phosphoproteomics (LFQ)	n.a.	Allows us to study the dynamics of phosphorylation. Global-scale identification of protein kinase targets. Unlimited number of samples can be compared.	Inhibitor might elicit off-target effects. Increased data variability. Increased instrument and computational time.

Note: n.a.—not applicable.

## References

[1] Hanks S.K., Hunter T. (1995). The eukaryotic protein kinase superfamily: Kinase (catalytic) domain structure and classification 1. FASEB J..

[2] Hunter T. (1995). Protein kinases and phosphatases: The Yin and Yang of protein phosphorylation and signaling. Cell.

[3] Manning G., Whyte D.B., Martinez R., Hunter T., Sudarsanam S. (2002). The Protein Kinase Complement of the Human Genome. Science.

[4] Carpy A., Krug K., Graf S., Koch A., Popic S., Hauf S., Macek B. (2014). Absolute Proteome and Phosphoproteome Dynamics during the Cell Cycle of Schizosaccharomyces pombe (Fission Yeast). Mol. Cell. Proteom..

[5] Chen Y., Nielsen J. (2016). Flux control through protein phosphorylation in yeast. FEMS Yeast Res..

[6] Salazar C., Höfer T. (2009). Multisite protein phosphorylation—From molecular mechanisms to kinetic models. FEBS J..

[7] Moura M., Conde C. (2019). Phosphatases in Mitosis: Roles and Regulation. Biomolecules.

[8] Ptacek J., Devgan G., Michaud G., Zhu H., Zhu X., Fasolo J., Guo H., Jona G., Breitkreutz A., Sopko R. (2005). Global analysis of protein phosphorylation in yeast. Nat. Cell Biol..

[9] Cohen-Armon M., Visochek L., Rozensal D., Kalal A., Geistrikh I., Klein R., Bendetz-Nezer S., Yao Z., Seger R. (2007). DNA-Independent PARP-1 Activation by Phosphorylated ERK2 Increases Elk1 Activity: A Link to Histone Acetylation. Mol. Cell.

[10] Scheeff E.D., Eswaran J., Bunkoczi G., Knapp S., Manning G. (2009). Structure of the Pseudokinase VRK3 Reveals a Degraded Catalytic Site, a Highly Conserved Kinase Fold, and a Putative Regulatory Binding Site. Structure.

[11] Liu L., Thaker T.M., Freed D.M., Frazier N., Malhotra K., Lemmon M.A., Jura N. (2018). Regulation of Kinase Activity in the Caenorhabditis elegans EGF Receptor, LET-23. Structure.

[12] Hu J., Yu H., Kornev A.P., Zhao J., Filbert E.L., Taylor S.S., Shaw A.S. (2011). Mutation that blocks ATP binding creates a pseudokinase stabilizing the scaffolding function of kinase suppressor of Ras, CRAF and BRAF. Proc. Natl. Acad. Sci. USA.

[13] Hu S., Xie Z., Onishi A., Yu X., Jiang L., Lin J., Rho H.-S., Woodard C., Wang H., Jeong J.-S. (2009). Profiling the Human Protein-DNA Interactome Reveals ERK2 as a Transcriptional Repressor of Interferon Signaling. Cell.

[14] Kollmann K., Heller G., Schneckenleithner C., Warsch W., Scheicher R., Ott R.G., Schäfer M., Fajmann S., Schlederer M., Schiefer A.-I. (2013). A Kinase-Independent Function of CDK6 Links the Cell Cycle to Tumor Angiogenesis. Cancer Cell.

[15] Lubelsky Y., Shaul Y. (2019). Recruitment of the protein phosphatase-1 catalytic subunit to promoters by the dual-function transcription factor RFX1. Biochem. Biophys. Res. Commun..

[16] Maddika S., Chen J. (2009). Protein kinase DYRK2 is a scaffold that facilitates assembly of an E3 ligase. Nat. Cell Biol..

[17] Murphy J.M., Nakatani Y., Jamieson S.A., Dai W., Lucet I.S., Mace P.D. (2015). Molecular Mechanism of CCAAT-Enhancer Binding Protein Recruitment by the TRIB1 Pseudokinase. Structure.

[18] Mikolaskova B., Jurcik M., Cipakova I., Kretova M., Chovanec M., Cipak L. (2018). Maintenance of genome stability: The unifying role of interconnections between the DNA damage response and RNA-processing pathways. Curr. Genet..

[19] Jacobsen A.V., Murphy J.M. (2017). The secret life of kinases: Insights into non-catalytic signalling functions from pseudokinases. Biochem. Soc. Trans..

[20] Kwon A., Scott S., Taujale R., Yeung W., Kochut K.J., Eyers P.A., Kannan N. (2019). Tracing the origin and evolution of pseudokinases across the tree of life. Sci. Signal..

[21] Kung J.E., Jura N. (2019). Prospects for pharmacological targeting of pseudokinases. Nat. Rev. Drug Discov..

[22] Schuler F., Weiss J.G., Lindner S.E., Lohmüller M., Herzog S., Spiegl S.F., Menke P., Geley S., Labi V., Villunger A. (2017). Checkpoint kinase 1 is essential for normal B cell development and lymphomagenesis. Nat. Commun..

[23] Sibanda B.L., Chirgadze D.Y., Ascher D.B., Blundell T.L. (2017). DNA-PKcs structure suggests an allosteric mechanism modulating DNA double-strand break repair. Science.

[24] Tiacci E., Pettirossi V., Schiavoni G., Falini B. (2017). Genomics of Hairy Cell Leukemia. J. Clin. Oncol..

[25] Corcoles-Saez I., Dong K., Cha R.S. (2019). Versatility of the Mec1ATM/ATR signaling network in mediating resistance to replication, genotoxic, and proteotoxic stresses. Curr. Genet..

[26] Way K.J., Katai N., King G.L. (2001). Protein kinase C and the development of diabetic vascular complications. Diabet. Med..

[27] Danielsson A., Öst A., Nyström F., Strålfors P. (2005). Attenuation of Insulin-stimulated Insulin Receptor Substrate-1 Serine 307 Phosphorylation in Insulin Resistance of Type 2 Diabetes. J. Biol. Chem..

[28] Lahiry P., Torkamani A., Schork N.J., Hegele R.A. (2010). Kinase mutations in human disease: Interpreting genotype–phenotype relationships. Nat. Rev. Genet..

[29] Wilson L.J., Linley A., Hammond D.E., Hood F.E., Coulson J.M., MacEwan D.J., Ross S.J., Slupsky J.R., Smith P.D., Eyers P.A. (2017). New Perspectives, Opportunities, and Challenges in Exploring the Human Protein Kinome. Cancer Res..

[30] Olsen J.V., Blagoev B., Gnad F., Macek B., Kumar C., Mortensen P., Mann M. (2006). Global, In Vivo, and Site-Specific Phosphorylation Dynamics in Signaling Networks. Cell.

[31] Besant P.G., Attwood P.V. (2005). Mammalian histidine kinases. Biochim. Biophys. Acta (BBA)—Proteins Proteom..

[32] Fuhs S.R., Meisenhelder J., Aslanian A., Ma L., Zagorska A., Stankova M., Binnie A., Al-Obeidi F., Mauger J., Lemke G. (2015). Monoclonal 1- and 3-Phosphohistidine Antibodies: New Tools to Study Histidine Phosphorylation. Cell.

[33] Hardman G., Perkins S., Brownridge P.J., Clarke C.J., Byrne D.P., Campbell A.E., Kalyuzhnyy A., Myall A., Eyers P.A., Jones A.R. (2019). Strong anion exchange-mediated phosphoproteomics reveals extensive human non-canonical phosphorylation. EMBO J..

[34] Sharma K., D’Souza R.C., Tyanova S., Schaab C., Wiśniewski J.R., Cox J., Mann M. (2014). Ultradeep Human Phosphoproteome Reveals a Distinct Regulatory Nature of Tyr and Ser/Thr-Based Signaling. Cell Rep..

[35] Beenstock J., Mooshayef N., Engelberg D. (2016). How Do Protein Kinases Take a Selfie (Autophosphorylate)?. Trends Biochem. Sci..

[36] Ahiri A., Garmes H., Podlipnik C., Aboulmouhajir A. (2019). Insights into evolutionary interaction patterns of the ‘Phosphorylation Activation Segment’ in kinase. Bioinformation.

[37] Combes G., Barysz H., Garand C., Braga L.G., Alharbi I., Thebault P., Murakami L., Bryne D.P., Stankovic S., Eyers P.A. (2018). Mps1 Phosphorylates Its N-Terminal Extension to Relieve Autoinhibition and Activate the Spindle Assembly Checkpoint. Curr. Biol..

[38] Pinna L., Ruzzene M. (1996). How do protein kinases recognize their substrates?. Biochim. et Biophys. Acta (BBA)—Bioenerg..

[39] Good M.C., Zalatan J.G., Lim W.A. (2011). Scaffold Proteins: Hubs for Controlling the Flow of Cellular Information. Science.

[40] Miller C.J., Turk B.E. (2018). Homing in: Mechanisms of Substrate Targeting by Protein Kinases. Trends Biochem. Sci..

[41] Shah N.H., Kuriyan J. (2019). Understanding molecular mechanisms in cell signaling through natural and artificial sequence variation. Nat. Struct. Mol. Biol..

[42] Gógl G., Kornev A.P., Reményi A., Taylor S.S. (2019). Disordered Protein Kinase Regions in Regulation of Kinase Domain Cores. Trends Biochem. Sci..

[43] Jenal U., Galperin M.Y. (2009). Single domain response regulators: Molecular switches with emerging roles in cell organization and dynamics. Curr. Opin. Microbiol..

[44] Schlessinger J. (2000). Cell Signaling by Receptor Tyrosine Kinases. Cell.

[45] Taylor S.S., Keshwani M.M., Steichen J.M., Kornev A.P. (2012). Evolution of the eukaryotic protein kinases as dynamic molecular switches. Philos. Trans. R. Soc. B: Biol. Sci..

[46] Taylor S.S., Kornev A.P. (2011). Protein kinases: Evolution of dynamic regulatory proteins. Trends Biochem. Sci..

[47] Kannan N., Wu J., Anand G.S., Yooseph S., Neuwald A.F., Venter J.C., Taylor S.S. (2007). Evolution of allostery in the cyclic nucleotide binding module. Genome Biol..

[48] Yang J., Kennedy E.J., Wu J., Deal M.S., Pennypacker J., Ghosh G., Taylor S.S. (2009). Contribution of Non-catalytic Core Residues to Activity and Regulation in Protein Kinase A. J. Biol. Chem..

[49] Lopez M.S., Kliegman J.I., Shokat K.M. (2014). The Logic and Design of Analog-Sensitive Kinases and Their Small Molecule Inhibitors. Methods in Enzymol..

[50] Huse M., Kuriyan J. (2002). The Conformational Plasticity of Protein Kinases. Cell.

[51] Kornev A.P., Taylor S.S. (2010). Defining the conserved internal architecture of a protein kinase. Biochim. Biophys. Acta (BBA)—Proteins Proteom..

[52] Iyer G.H., Garrod S., Woods V.L., Taylor S.S. (2005). Catalytic Independent Functions of a Protein Kinase as Revealed by a Kinase-dead Mutant: Study of the Lys72His Mutant of cAMP-dependent Kinase. J. Mol. Biol..

[53] Iyer G.H., Moore M.J., Taylor S.S. (2005). Consequences of Lysine 72 Mutation on the Phosphorylation and Activation State of cAMP-dependent Kinase. J. Biol. Chem..

[54] Ling P., Yao Z., Meyer C.F., Wang X.S., Oehrl W., Feller S.M., Tan T.-H. (1999). Interaction of Hematopoietic Progenitor Kinase 1 with Adapter Proteins Crk and CrkL Leads to Synergistic Activation of c-Jun N-Terminal Kinase. Mol. Cell. Biol..

[55] Petronczki M., Matos J., Mori S., Gregan J., Bogdanova A., Schwickart M., Mechtler K., Shirahige K., Zachariae W., Nasmyth K. (2006). Monopolar Attachment of Sister Kinetochores at Meiosis I Requires Casein Kinase 1. Cell.

[56] Force T., Kuida K., Namchuk M., Parang K., Kyriakis J.M. (2004). Inhibitors of Protein Kinase Signaling Pathways. Circulation.

[57] Bhullar K.S., Lagarón N.O., McGowan E.M., Parmar I., Jha A., Hubbard B.P., Rupasinghe H.P.V. (2018). Kinase-targeted cancer therapies: Progress, challenges and future directions. Mol. Cancer.

[58] Bishop A.C., Shah K., Liu Y., Witucki L., Kung C.-Y., Shokat K.M. (1998). Design of allele-specific inhibitors to probe protein kinase signaling. Curr. Biol..

[59] Islam K. (2018). The Bump-and-Hole Tactic: Expanding the Scope of Chemical Genetics. Cell Chem. Biol..

[60] Hanke J.H., Gardner J.P., Dow R.L., Changelian P.S., Brissette W.H., Weringer E.J., Pollok B.A., Connelly P.A. (1996). Discovery of a novel, potent, and Src family-selective tyrosine kinase inhibitor. Study of Lck- and FynT-dependent T cell activation. J. Biol. Chem..

[61] Liu Y., Bishop A., Witucki L., Kraybill B., Shimizu E., Tsien J., Ubersax J., Blethrow J., Morgan D.O., Shokat K.M. (1999). Structural basis for selective inhibition of Src family kinases by PP1. Chem. Biol..

[62] Witucki L.A., Huang X., Shah K., Liu Y., Kyin S., Eck M.J., Shokat K.M. (2002). Mutant Tyrosine Kinases with Unnatural Nucleotide Specificity Retain the Structure and Phospho-Acceptor Specificity of the Wild-Type Enzyme. Chem. Biol..

[63] Gum R.J. (1998). Acquisition of Sensitivity of Stress-activated Protein Kinases to the p38 Inhibitor, SB 203580, by Alteration of One or More Amino Acids within the ATP Binding Pocket. J. Biol. Chem..

[64] Eyers P.A., Craxton M., Morricel N., Cohen P., Goedert M. (1998). Conversion of SB 203580-insensitive MAP kinase family members to drug-sensitive forms by a single amino-acid substitution. Chem. Biol..

[65] Shah K., Liu Y., Deirmengian C., Shokat K.M. (1997). Engineering unnatural nucleotide specificity for Rous sarcoma virus tyrosine kinase to uniquely label its direct substrates. Proc. Natl. Acad. Sci. USA.

[66] Shi F., Telesco S.E., Liu Y., Radhakrishnan R., Lemmon M.A. (2010). ErbB3/HER3 intracellular domain is competent to bind ATP and catalyze autophosphorylation. Proc. Natl. Acad. Sci. USA.

[67] Bishop A.C., Ubersax J.A., Petsch D.T., Matheos D.P., Gray N.S., Blethrow J., Shimizu E., Tsien J.Z., Schultz P.G., Rose M.D. (2000). A chemical switch for inhibitor-sensitive alleles of any protein kinase. Nature.

[68] Cipak L., Zhang C., Kovacikova I., Rumpf C., Miadoková E., Shokat K.M., Gregan J. (2011). Generation of a set of conditional analog-sensitive alleles of essential protein kinases in the fission yeast Schizosaccharomyces pombe. Cell Cycle.

[69] Gregan J., Zhang C., Rumpf C., Cipak L., Li Z., Uluocak P., Nasmyth K., Shokat K.M. (2007). Construction of conditional analog-sensitive kinase alleles in the fission yeast Schizosaccharomyces pombe. Nat. Protoc..

[70] Nolen B., Taylor S., Ghosh G. (2004). Regulation of protein kinases; controlling activity through activation segment conformation. Mol. Cell.

[71] Fabbro D., Cowan-Jacob S.W., Moebitz H. (2015). Ten things you should know about protein kinases: IUPHAR Review 14. Br. J. Pharmacol..

[72] Bailey F.P., Andreev V.I., Eyers P.A. (2014). The Resistance Tetrad. Methods Enzymol..

[73] Persky N.S., Hernandez D., Carmo M.D., Brenan L., Cohen O., Kitajima S., Nayar U., Walker A., Pantel S., Lee Y. (2020). Defining the landscape of ATP-competitive inhibitor resistance residues in protein kinases. Nat. Struct. Mol. Biol..

[74] Gorre M.E., Mohammed M., Ellwood K., Hsu N., Paquette R., Rao P.N., Sawyers C.L. (2001). Clinical Resistance to STI-571 Cancer Therapy Caused by BCR-ABL Gene Mutation or Amplification. Science.

[75] Kobayashi S., Boggon T.J., Dayaram T., Jänne P.A., Kocher O., Meyerson M., Johnson B.E., Eck M.J., Tenen D.G., Halmos B. (2005). EGFR mutation and resistance of non-small-cell lung cancer to gefitinib. N. Engl. J. Med..

[76] Romano V., De Beer T.A.P., Schwede T. (2017). A computational protocol to evaluate the effects of protein mutants in the kinase gatekeeper position on the binding of ATP substrate analogues. BMC Res. Notes.

[77] Lera R.F., Burkard M.E. (2012). The Final Link: Tapping the Power of Chemical Genetics to Connect the Molecular and Biologic Functions of Mitotic Protein Kinases. Molecules.

[78] Fleißner A. (2013). Turning the switch: Using chemical genetics to elucidate protein kinase functions in filamentous fungi. Fungal Biol. Rev..

[79] Aoi Y., Kawashima S.A., Simanis V., Yamamoto M., Sato M. (2014). Optimization of the analogue-sensitive Cdc2/Cdk1 mutant by in vivo selection eliminates physiological limitations to its use in cell cycle analysis. Open Biol..

[80] Swaffer M.P., Jones A.W., Flynn H.R., Snijders A.P., Nurse P. (2016). CDK Substrate Phosphorylation and Ordering the Cell Cycle. Cell.

[81] Swaffer M.P., Jones A.W., Flynn H.R., Snijders A.P., Nurse P. (2018). Quantitative Phosphoproteomics Reveals the Signaling Dynamics of Cell-Cycle Kinases in the Fission Yeast Schizosaccharomyces pombe. Cell Rep..

[82] Bishop A.C., Buzko O., Shokat K.M. (2001). Magic bullets for protein kinases. Trends Cell Biol..

[83] Kenski D.M., Zhang C., Von Zastrow M., Shokat K.M. (2005). Chemical Genetic Engineering of G Protein-coupled Receptor Kinase 2. J. Biol. Chem..

[84] Zhang C., Kenski D.M., Paulson J.L., Bonshtien A., Sessa G., Cross J.V., Templeton D.J., Shokat K.M. (2005). A second-site suppressor strategy for chemical genetic analysis of diverse protein kinases. Nat. Methods.

[85] Schindler T., Sicheri F., Pico A.R., Gazit A., Levitzki A., Kuriyan J. (1999). Crystal Structure of Hck in Complex with a Src Family–Selective Tyrosine Kinase Inhibitor. Mol. Cell.

[86] Weiss E.L., Bishop A.C., Shokat K.M., Drubin D.G. (2000). Chemical genetic analysis of the budding-yeast p21-activated kinase Cla4p. Nat. Cell Biol..

[87] Sekiya-Kawasaki M., Groen A.C., Cope M.J.T., Kaksonen M., Watson H.A., Zhang C., Shokat K.M., Wendland B., McDonald K.L., McCaffery J.M. (2003). Dynamic phosphoregulation of the cortical actin cytoskeleton and endocytic machinery revealed by real-time chemical genetic analysis. J. Cell Biol..

[88] Wan L., Zhang C., Shokat K.M., Hollingsworth N.M. (2006). Chemical Inactivation of Cdc7 Kinase in Budding Yeast Results in a Reversible Arrest That Allows Efficient Cell Synchronization Prior to Meiotic Recombination. Genetics.

[89] Johnson E.O., Chang K.-H., Ghosh S., Venkatesh C., Giger K., Low P.S., Shah K. (2012). LIMK2 is a crucial regulator and effector of Aurora-A-kinase-mediated malignancy. J. Cell Sci..

[90] Blethrow J., Zhang C., Shokat K.M., Weiss E.L. (2004). Design and Use of Analog-Sensitive Protein Kinases. Curr. Protoc. Mol. Biol..

[91] Kung C., Kenski D.M., Dickerson S.H., Howson R.W., Kuyper L.F., Madhani H.D., Shokat K.M. (2005). Chemical genomic profiling to identify intracellular targets of a multiplex kinase inhibitor. Proc. Natl. Acad. Sci. USA.

[92] Guo T., Marmol P., Moliner A., Björnholm M., Zhang C., Shokat K.M., Ibáñez C.F. (2014). Adipocyte ALK7 links nutrient overload to catecholamine resistance in obesity. eLife.

[93] Grallert A., Patel A., Tallada V.A., Chan K.Y., Bagley S., Krapp A., Simanis V., Hagan I.M. (2012). Centrosomal MPF triggers the mitotic and morphogenetic switches of fission yeast. Nat. Cell Biol..

[94] Tay Y.D., Patel A., Kaemena D.F., Hagan I.M. (2013). Mutation of a conserved residue enhances the sensitivity of analogue-sensitised kinases to generate a novel approach to the study of mitosis in fission yeast. J. Cell Sci..

[95] Ocasio C.A., Warkentin A.A., McIntyre P.J., Barkovich K.J., Vesely C., Spencer J., Shokat K.M., Bayliss R. (2018). Type II Kinase Inhibitors Targeting Cys-Gatekeeper Kinases Display Orthogonality with Wild Type and Ala/Gly-Gatekeeper Kinases. ACS Chem. Biol..

[96] Baltussen L.L., Negraes P.D., Silvestre M., Claxton S., Moeskops M., Christodoulou E., Flynn H., Snijders A.P., Muotri A.R., Ultanir S.K. (2018). Chemical genetic identification of CDKL 5 substrates reveals its role in neuronal microtubule dynamics. EMBO J..

[97] Muñoz I.M., Morgan M.E., Peltier J., Weiland F., Gregorczyk M., Brown F.C., Macartney T., Toth R., Trost M., Rouse J. (2018). Phosphoproteomic screening identifies physiological substrates of the CDKL 5 kinase. EMBO J..

[98] Fan Z., Devlin J.R., Hogg S.J., Doyle M.A., Harrison P.F., Todorovski I., Cluse L.A., Knight D.A., Sandow J.J., Gregory G.P. (2020). CDK13 cooperates with CDK12 to control global RNA polymerase II processivity. Sci. Adv..

[99] Hernández-Ortega S., Sánchez-Botet A., Quandt E., Masip N., Gasa L., Verde G., Jiménez J., Levin R.S., Rutaganira F.U., Burlingame A.L. (2019). Phosphoregulation of the oncogenic protein regulator of cytokinesis 1 (PRC1) by the atypical CDK16/CCNY complex. Exp. Mol. Med..

[100] Ferguson F.M., Gray N.S. (2018). Kinase inhibitors: The road ahead. Nat. Rev. Drug Discov..

[101] Shah K., Kim H. (2019). The significant others: Global search for direct kinase substrates using chemical approaches. IUBMB Life.

[102] Needham E.J., Parker B.L., Burykin T., James D.E., Humphrey S.J. (2019). Illuminating the dark phosphoproteome. Sci. Signal..

[103] Ficarro S.B., McCleland M.L., Stukenberg P.T., Burke D.J., Ross M.M., Shabanowitz J., Hunt D.F., White F.M. (2002). Phosphoproteome analysis by mass spectrometry and its application to Saccharomyces cerevisiae. Nat. Biotechnol..

[104] Beausoleil S.A., Jedrychowski M., Schwartz D., Elias J.E., Villén J., Li J., Cohn M.A., Cantley L.C., Gygi S.P. (2004). Large-scale characterization of HeLa cell nuclear phosphoproteins. Proc. Natl. Acad. Sci. USA.

[105] Blagoev B., Ong S.-E., Kratchmarova I., Mann M. (2004). Temporal analysis of phosphotyrosine-dependent signaling networks by quantitative proteomics. Nat. Biotechnol..

[106] Humphrey S.J., James D.E., Mann M. (2015). Protein Phosphorylation: A Major Switch Mechanism for Metabolic Regulation. Trends Endocrinol. Metab..

[107] Fíla J., Honys D. (2011). Enrichment techniques employed in phosphoproteomics. Amino Acids.

[108] Iliuk A., Jayasundera K., Wang W.-H., Schluttenhofer R., Geahlen R.L., Tao W.A. (2015). In-depth analyses of B cell signaling through tandem mass spectrometry of phosphopeptides enriched by PolyMAC. Int. J. Mass Spectrom..

[109] Cao Z., Tang H.-Y., Wang H., Liu Q., Speicher D.W. (2012). Systematic Comparison of Fractionation Methods for In-depth Analysis of Plasma Proteomes. J. Proteome Res..

[110] Alpert A.J. (2008). Electrostatic Repulsion Hydrophilic Interaction Chromatography for Isocratic Separation of Charged Solutes and Selective Isolation of Phosphopeptides. Anal. Chem..

[111] Wirbel J., Cutillas P., Saez-Rodriguez J. (2018). Phosphoproteomics-Based Profiling of Kinase Activities in Cancer Cells. Methods Mol. Biol..

[112] Keller A., Bader S.L., Kusebauch U., Shteynberg D., Hood L., Moritz R.L. (2015). Opening a SWATH Window on Posttranslational Modifications: Automated Pursuit of Modified Peptides. Mol. Cell. Proteom..

[113] Riley N.M., Coon J.J. (2016). Phosphoproteomics in the Age of Rapid and Deep Proteome Profiling. Anal. Chem..

[114] Bodenmiller B., Wanka S., Kraft C., Urban J., Campbell D., Pedrioli P.G., Gerrits B., Picotti P., Lam H., Vitek O. (2010). Phosphoproteomic Analysis Reveals Interconnected System-Wide Responses to Perturbations of Kinases and Phosphatases in Yeast. Sci. Signal..

[115] Shi T., Yao L., Han Y., Hao P., Lu P. (2019). Quantitative Phosphoproteomics Reveals System-Wide Phosphorylation Network Altered by Spry in Mouse Mammary Stromal Fibroblasts. Int. J. Mol. Sci..

[116] Newman R.H., Zhang J. (2017). Integrated Strategies to Gain a Systems-Level View of Dynamic Signaling Networks. Methods Enzym..

[117] Newman R.H., Zhang J., Zhu H. (2014). Toward a systems-level view of dynamic phosphorylation networks. Front. Genet..

[118] Tien A.-C., Lin M.-H., Su L.-J., Hong Y.-R., Cheng T.-S., Lee Y.-C.G., Lin W.-J., Still I.H., Huang C.-Y.F. (2004). Identification of the Substrates and Interaction Proteins of Aurora Kinases from a Protein-Protein Interaction Model. Mol. Cell. Proteom..

[119] Amano M., Tsumura Y., Taki K., Harada H., Mori K., Nishioka T., Kato K., Suzuki T., Nishioka Y., Iwamatsu A. (2010). A Proteomic Approach for Comprehensively Screening Substrates of Protein Kinases Such as Rho-Kinase. PLoS ONE.

[120] Daub H., Olsen J.V., Bairlein M., Gnad F., Oppermann F.S., Körner R., Greff Z., Kéri G., Stemmann O., Mann M. (2008). Kinase-Selective Enrichment Enables Quantitative Phosphoproteomics of the Kinome across the Cell Cycle. Mol. Cell.

[121] Belozerov V.E., Lin Z.-Y., Gingras A., McDermott J.C., Siu K.W.M. (2012). High-Resolution Protein Interaction Map of the Drosophila melanogaster p38 Mitogen-Activated Protein Kinases Reveals Limited Functional Redundancy. Mol. Cell. Biol..

[122] Phadnis N., Cipak L., Polakova S., Hyppa R.W., Cipakova I., Anrather D., Karvaiova L., Mechtler K., Smith G.R., Gregan J. (2015). Casein Kinase 1 and Phosphorylation of Cohesin Subunit Rec11 (SA3) Promote Meiotic Recombination through Linear Element Formation. PLoS Genet..

[123] Cann M.L., McDonald I.M., East M.P., Johnson G.L., Graves L.M. (2017). Measuring Kinase Activity-A Global Challenge. J. Cell. Biochem..

[124] Delom F., Chevet E. (2006). Phosphoprotein analysis: From proteins to proteomes. Proteome Sci..

[125] Goodwin C.R., Woodard C.L., Zhou X., Pan J., Olivi A., Xia S., Bettegowda C., Sciubba D.M., Pevsner J., Zhu H. (2016). Microarray-Based Phospho-Proteomic Profiling of Complex Biological Systems12. Transl. Oncol..

[126] Amano M., Hamaguchi T., Shohag H., Kozawa K., Kato K., Zhang X., Yura Y., Matsuura Y., Kataoka C., Nishioka T. (2015). Kinase-interacting substrate screening is a novel method to identify kinase substrates. J. Cell Biol..

[127] Amano M., Nishioka T., Yura Y., Kaibuchi K. (2016). Identification of Protein Kinase Substrates by the Kinase-Interacting Substrate Screening (KISS) Approach. Curr. Protoc. Cell Biol..

[128] Müller A.C., Giambruno R., Weißer J., Májek P., Hofer A., Bigenzahn J.W., Superti-Furga G., Jessen H., Bennett K.L. (2016). Identifying Kinase Substrates via a Heavy ATP Kinase Assay and Quantitative Mass Spectrometry. Sci. Rep..

[129] Nishioka T., Shohag H., Amano M., Kaibuchi K. (2015). Developing novel methods to search for substrates of protein kinases such as Rho-kinase. Biochim. Biophys. Acta (BBA)—Proteins Proteom..

[130] Obsil T., Obsilova V. (2011). Structural basis of 14-3-3 protein functions. Semin. Cell Dev. Biol..

[131] Sluchanko N.N. (2020). Reading the phosphorylation code: Binding of the 14-3-3 protein to multivalent client phosphoproteins. Biochem. J..

[132] Moritz A., Li Y., Guo A., Villén J., Wang Y., MacNeill J., Kornhauser J., Sprott K., Zhou J., Possemato A. (2010). Akt-RSK-S6 Kinase Signaling Networks Activated by Oncogenic Receptor Tyrosine Kinases. Sci. Signal..

[133] Grønborg M., Kristiansen T.Z., Stensballe A., Andersen J.S., Ohara O., Mann M., Jensen O.N., Pandey A. (2002). A mass spectrometry-based proteomic approach for identification of serine/threonine-phosphorylated proteins by enrichment with phospho-specific antibodies: Identification of a novel protein, Frigg, as a protein kinase A substrate. Mol. Cell. Proteom..

[134] Shohag H., Nishioka T., Ahammad R.U., Nakamuta S., Yura Y., Hamaguchi T., Kaibuchi K., Amano M. (2015). Phosphoproteomic Analysis Using the WW and FHA Domains as Biological Filters. Cell Struct. Funct..

[135] Nagai T., Yoshimoto J., Kannon T., Kuroda K., Kaibuchi K. (2016). Phosphorylation Signals in Striatal Medium Spiny Neurons. Trends Pharmacol. Sci..

[136] Nishioka T., Amano M., Funahashi Y., Tsuboi D., Yamahashi Y., Kaibuchi K. (2019). In Vivo Identification of Protein Kinase Substrates by Kinase-Oriented Substrate Screening (KIOSS). Curr. Protoc. Chem. Biol..

[137] Tsai C.-F., Wang Y.-T., Yen H.-Y., Tsou C.-C., Ku W.-C., Lin P.-Y., Chen H.-Y., Nesvizhskii A.I., Ishihama Y., Chen Y.-J. (2015). Large-scale determination of absolute phosphorylation stoichiometries in human cells by motif-targeting quantitative proteomics. Nat. Commun..

[138] Ma W., Zhang D., Li G., Liu J., He G., Zhang P., Yang L., Zhu H.-X., Xu N., Liang S. (2017). Antibacterial mechanism of daptomycin antibiotic against Staphylococcus aureus based on a quantitative bacterial proteome analysis. J. Proteom..

[139] Yang Y., He Y., Wang X., Liang Z., He G., Zhang P., Zhu H., Xu N., Liang S. (2017). Protein SUMOylation modification and its associations with disease. Open Biol..

[140] Byrne D.P., Clarke C.J., Brownridge P.J., Kalyuzhnyy A., Perkins S., Campbell A., Mason D., Jones A.R., Eyers P.A., Eyers C.E. (2020). Use of the Polo-like kinase 4 (PLK4) inhibitor centrinone to investigate intracellular signaling networks using SILAC-based phosphoproteomics. Biochem. J..

[141] Krapp A., Hamelin R., Armand F., Chiappe D., Krapp L., Cano E., Moniatte M., Simanis V. (2019). Analysis of the S. pombe Meiotic Proteome Reveals a Switch from Anabolic to Catabolic Processes and Extensive Post-transcriptional Regulation. Cell Rep..

[142] Huraiova B., Kanovits J., Polakova S.B., Cipak L., Benko Z., Sevcovicova A., Anrather D., Ammerer G., Duncan C.D.S., Mata J. (2020). Proteomic analysis of meiosis and characterization of novel short open reading frames in the fission yeast Schizosaccharomyces pombe. Cell Cycle.

[143] Liang S., Yu Y., Yang P., Gu S., Xue Y., Chen X. (2009). Analysis of the protein complex associated with 14-3-3 epsilon by a deuterated-leucine labeling quantitative proteomics strategy. J. Chromatogr. B Analyt. Technol. Biomed. Life Sci..

[144] Kettenbach A.N., Deng L., Wu Y., Baldissard S., Adamo M.E., Gerber S.A., Moseley J.B. (2015). Quantitative Phosphoproteomics Reveals Pathways for Coordination of Cell Growth and Division by the Conserved Fission Yeast Kinase Pom1. Mol. Cell. Proteom..

[145] Geiger T., Wisniewski J.R., Cox J., Zanivan S., Kruger M., Ishihama Y., Mann M. (2011). Use of stable isotope labeling by amino acids in cell culture as a spike-in standard in quantitative proteomics. Nat. Protoc..

[146] Hilger M., Mann M. (2011). Triple SILAC to Determine Stimulus Specific Interactions in the Wnt Pathway. J. Proteome Res..

[147] Geiger T., Cox J., Ostasiewicz P., Wisniewski J.R., Mann M. (2010). Super-SILAC mix for quantitative proteomics of human tumor tissue. Nat. Methods.

[148] Ross P.L., Huang Y.N., Marchese J.N., Williamson B., Parker K., Hattan S., Khainovski N., Pillai S., Dey S., Daniels S. (2004). Multiplexed Protein Quantitation inSaccharomyces cerevisiaeUsing Amine-reactive Isobaric Tagging Reagents. Mol. Cell. Proteom..

[149] Shiio Y., Aebersold R. (2006). Quantitative proteome analysis using isotope-coded affinity tags and mass spectrometry. Nat. Protoc..

[150] Goshe M.B., Conrads T.P., Panisko E.A., Angell N.H., Veenstra T.D., Smith R.D. (2001). Phosphoprotein Isotope-Coded Affinity Tag Approach for Isolating and Quantitating Phosphopeptides in Proteome-Wide Analyses. Anal. Chem..

[151] Qian W.-J., Goshe M.B., Camp D.G., Yu L.-R., Tang K., Smith R.D. (2003). Phosphoprotein Isotope-Coded Solid-Phase Tag Approach for Enrichment and Quantitative Analysis of Phosphopeptides from Complex Mixtures. Anal. Chem..

[152] Thompson A., Schäfer J., Kuhn K., Kienle S., Schwarz J., Schmidt G., Neumann A.T., Hamon C. (2003). Tandem Mass Tags: A Novel Quantification Strategy for Comparative Analysis of Complex Protein Mixtures by MS/MS. Anal. Chem..

[153] Hogrebe A., Von Stechow L., Bekker-Jensen D.B., Weinert B.T., Kelstrup C.D., Olsen J.V. (2018). Benchmarking common quantification strategies for large-scale phosphoproteomics. Nat. Commun..

[154] Aebersold R., Mann M. (2003). Mass spectrometry-based proteomics. Nat. Cell Biol..

[155] Ramus C., Hovasse A., Marcellin M., Hesse A.-M., Mouton-Barbosa E., Bouyssié D., Vaca S., Carapito C., Chaoui K., Bruley C. (2016). Benchmarking quantitative label-free LC–MS data processing workflows using a complex spiked proteomic standard dataset. J. Proteom..

[156] Huang T., Bruderer R., Muntel J., Xuan Y., Vitek O., Reiter L. (2019). Combining Precursor and Fragment Information for Improved Detection of Differential Abundance in Data Independent Acquisition. Mol. Cell. Proteom..

